# Sexual and reproductive health knowledge, sexual attitudes, and sexual behaviour of university students: Findings of a Beijing-Based Survey in 2010-2011

**DOI:** 10.1186/s13690-021-00739-5

**Published:** 2021-11-29

**Authors:** Ming Guan

**Affiliations:** 1 International Issues Center, Xuchang University, Road Bayi 88, Xuchang, Henan China; 2 Family Issues Center, Xuchang University, Road Bayi 88, Xuchang, Henan China; 3 School of Business, Xuchang University, Road Bayi 88, Xuchang, Henan China

**Keywords:** sexual attitudes, sexual behaviors, sources and categories of SRH knowledge, categories of contraceptive knowledge, mediating relationship, confounding effects, structural equation modeling

## Abstract

**Background:**

Although several studies have attempted investigating sex-related knowledge, attitudes, and practices among young people in China, deeper insights were still needed to further understand how this population could be supported to make healthy and safe sexual choices. Against this background, this study used a large set of secondary data to examine associations among sexual and reproductive health (SRH) knowledge, sexual attitudes, and sexual behaviour.

**Methods:**

A cross-sectional study was employed to explore the associations among SRH knowledge, sexual attitudes, and sexual behaviour with a publicly available survey data among the 1196 university students from freshmen to Ph.D. candidates. Descriptive analysis was used to describe the sociodemographic characteristics of the university students by gender. Associations of sociodemographic factors with sources and categories of SRH knowledge, categories of contraceptive knowledge, sexual attitudes, and sexual behavior were explored with Poisson regressions and logistic regressions, respectively. The mediating effects of sociodemographic factors on the associations between SRH knowledge and sexual behavior, observed sexual attitudes on the associations of SRH knowledge with sexual behavior, and latent sexual attitudes on the relationship between SRH knowledge and sexual behavior were analyzed in order.

**Results:**

Descriptive analysis showed that the sample was dominated by age group (18 to 24 years), undergraduates, females, limited contraceptive knowledge, unfavorable sexual attitudes, and insufficient knowledge sources. Regression analyses showed that sociodemographic factors had significant associations with SRH knowledge, sexual attitudes, and sexual behavior. Subsequently, the mediating effects of sociodemographic factors on the associations of SRH knowledge and sexual attitudes with sexual behavior were confirmed. Controlling for sociodemographic factors, the effects of sexual attitudes on the associations between SRH knowledge and sexual behaviour could be verified. Structural equation modeling indicated that the linear sequence of sources and categories of SRH knowledge → sexual attitudes → sexual behaviour model and the triangle mediating effects of sexual behaviour → sexual attitudes → SRH knowledge model existed.

**Conclusions:**

Sociodemographic factors and observed sexual attitudes mediated the associations between SRH knowledge and sexual behaviour. The sequence relationship: sources and categories of SRH knowledge → sexual attitudes → sexual behavior and the mediating relationship: sexual behavior → sexual attitude → sources and categories of SRH knowledge & sexual behavior → sources and categories of SRH knowledge and sexual behavior → sexual attitude → categories of contraceptive knowledge & sexual behavior → categories of contraceptive knowledge were confirmed in the sample. This study also identified an urgent need for the university students to access to SRH comprehensive knowledge.

**Supplementary Information:**

The online version contains supplementary material available at 10.1186/s13690-021-00739-5.

## Background

Young people remain a priority population for sexual and reproductive health (SRH) concerns as they continuously account for higher proportions of sexually transmissible infections (STIs) and risky sexual behaviour than other population groups. But, limited knowledge of emergency contraception among university students was reported [1–3]. Moreover, some studies revealed that individual differences in religiosity domains [4], school and parents [5], and sexual behavior of peers [6] influenced adolescents' sexual attitudes and behavioral intent. Till now in China, few studies reflected the possible associations of sociodemographic factors with SRH knowledge, sexual attitudes, and sexual behaviour.

An impressive body of empirical research indicated that insufficient or inaccurate SRH knowledge could lead to negative health outcomes. For example, a study confirmed that inappropriate SRH knowledge might obstruct problematic sexual health behavioral [7]. Without emergency contraception knowledge, risks for unintended pregnancy and reproductive tract infections might be increasing. For example, a study reported that sex without condoms contributed to premarital pregnancy among female undergraduates in China [8]. Additionally, a small but growing body of empirical research revealed unprotected sex in adolescents [9, 10] and its adverse effects included depressive symptoms [11] and sexual victimization [12]. To date, the possible relationship between SRH knowledge and sexual behaviour of university students in China was seldom reported.

Several studies indicated that the sexual attitudes could produce change in sexual behavioral among the university students. For example, attitudes towards contraception during adolescence were likely to cause adult contraceptive behavior [13]. Likewise, college-educated women were significantly more likely to transition from sexual relationships to cohabiting unions into marriage than less-educated women [14]. In particular, a study suggested that psychological difference between cohabiting and married individuals was confirmed [15]. As to the issues in China, the possible relationship between sexual attitudes and sexual behaviour of university students was needed to further studied.

Some other studies reported the relationship between sexual behavioral and SRH knowledge. For example, knowledge concerning STIs and the risks was insufficient in female Chinese college students [16]. A significant percentage of the college students have poor knowledge, attitude, and practice toward risky sexual behaviors in southwest Ethiopia [17]. Also, the relationship between SRH knowledge and sexual attitudes was documented. For example, a study among school going adolescents reported varied perceptions towards sex education [18]. Regarding these topics in China, the possible associations among SRH knowledge, sexual attitudes, and sexual behaviour were vital to persons accepting higher education.

The aims of this study were to assess the confounding effects of sociodemographic factors on SRH knowledge (sources and categories of SRH knowledge and categories of contraceptive knowledge), sexual attitudes, and sexual behavioral and to determine associations among SRH knowledge, sexual attitudes, and sexual behavioral among the university students with a publicly available survey data. In this study, a series of regressions, mediating analyses, and structural equation models were designed to provide evidences for the associations above. The empirical findings from statistical analyses would provide insights for policy interventions in sexual education for university students.

## Literature review

Knowledge, attitude, and behaviour are vital in the fields of sexual health education. The confounding effects of sociodemographic factors on SRH knowledge, sexual attitudes, and sexual behaviour were documented. For example, age, education, and wealth were significant associated with increase in STIs knowledge [19]. The relevant findings were published globally. Inadequate SRH information were reported among refugee adolescent girls in Uganda [20], Kurdish Syrian refugee young women living in Lebanon [21], Mozambican families [22], and adolescent mothers from indigenous populations in Cambodia [23]. Another study among Saudi males indicated that age, education, and income had high impact on contraception awareness and utilization [24]. Consistent with literature, this study therefore hypothesized that:

H0: Sociodemographic factors have significant associations with SRH knowledge, sexual attitudes, and sexual behaviour in China’ settings.

The relationships among SRH knowledge, sexual attitudes, and sexual behaviour were documented. For example, a correlation between knowledge, attitude and behavior was confirmed in medical training [25, 26]. Regarding the relationship between sexual knowledge and behaviour, another study indicated that poor SRH knowledge of male youths increased the risk of female partners’ unintended pregnancy in China [27]. Regarding the relationship between sexual attitude and behaviour, a study concluded that psychological and social factors could jointly reduce sexual risk behaviours [28]. Furthermore, several psychosocial predictors of high-risk sexual behaviour were identified [29]. The findings in a nutrition study suggest that gamification dissemination of knowledge could lead to behavioural improvements of adolescents [30]. Clinically, a 'knowledge-attitude-behaviour' sequence was suggested for physician to incorporate clinical practice guidelines into their practice [31]. Similarly, poor level and sources SRH knowledge and perceptions exposed young men to poor sexual outcomes [32]. Accordingly, this study hypothesized that:

H1: The linear sequence of SRH knowledge → sexual attitude → sexual behavior may exist.

H2: The triangle mediating effect of SRH knowledge → sexual attitude → sexual behavior may exist.

**Fig. 1:**
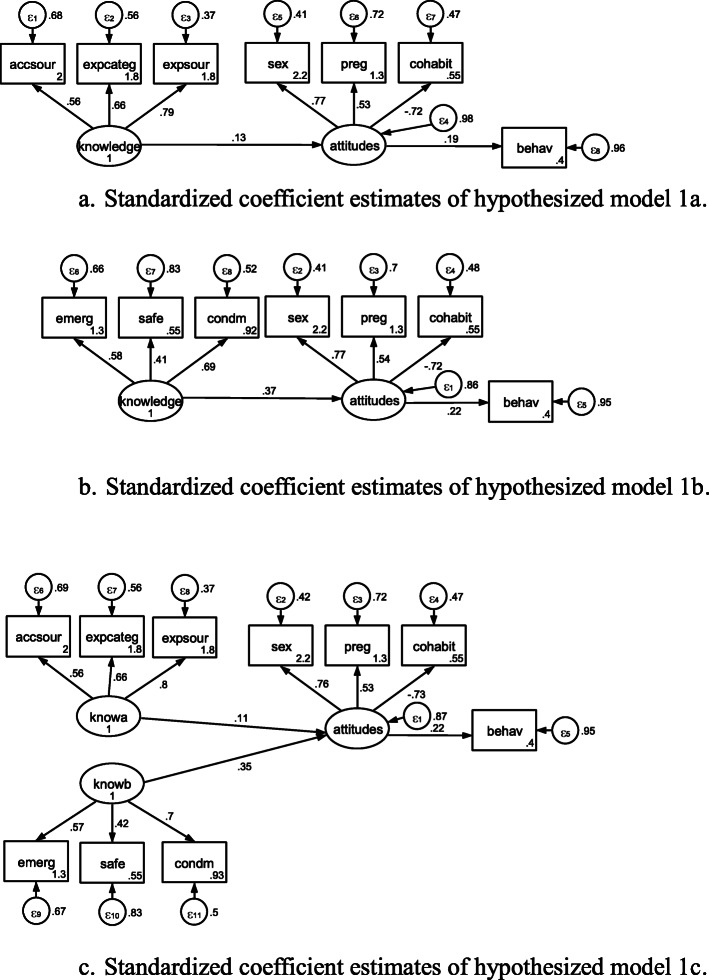
**a**. Standardized coefficient estimates of hypothesized model 1a. **b**. Standardized coefficient estimates of hypothesized model 1b. **c**. Standardized coefficient estimates of hypothesized model 1c.

**Fig. 2:**
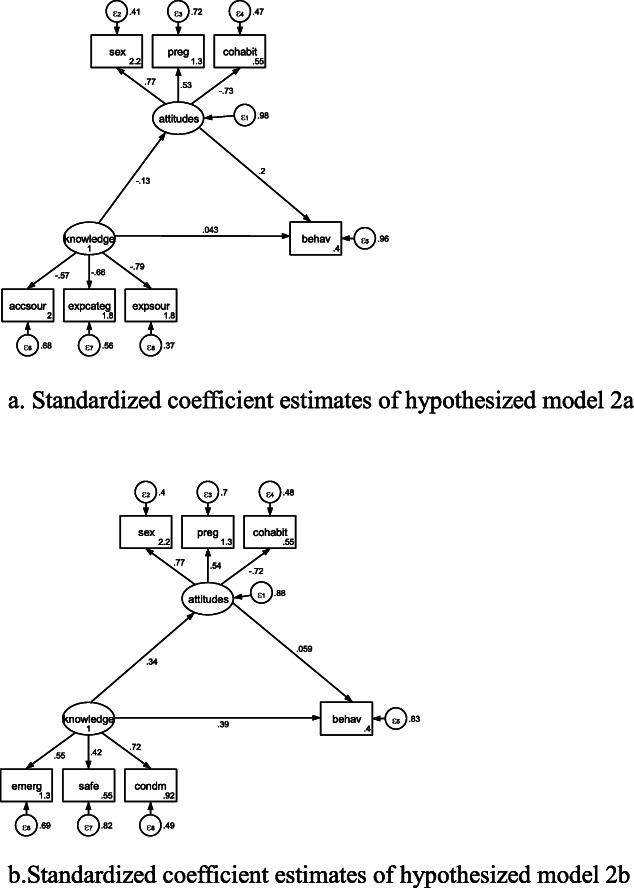
**a**. Standardized coefficient estimates of hypothesized model 2a. **b**. Standardized coefficient estimates of hypothesized model 2b

Thus, hypothesized Figures 1 and 2 could be depicted as follow:


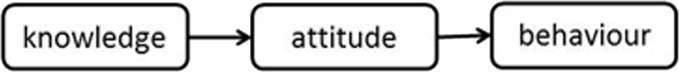


Hypothesized Figure 1. Linear sequence model.


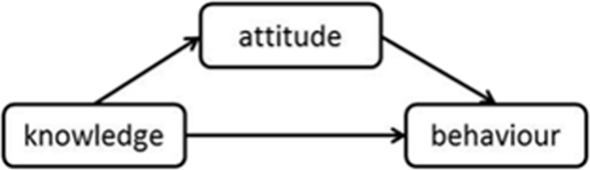


Hypothesized Figure 2. Triangle mediating model.

Combining the logic presented in the rationale for Hypotheses 1 and 2, the Hypotheses with reverse direction could be designed and were depicted as Hypotheses 3 and 4:

H3: The linear sequence of sexual behaviour → sexual attitude → SRH knowledge may exist.

H4: The triangle mediating effect of sexual behaviour → sexual attitude → SRH knowledge may exist.

**Fig. 3:**
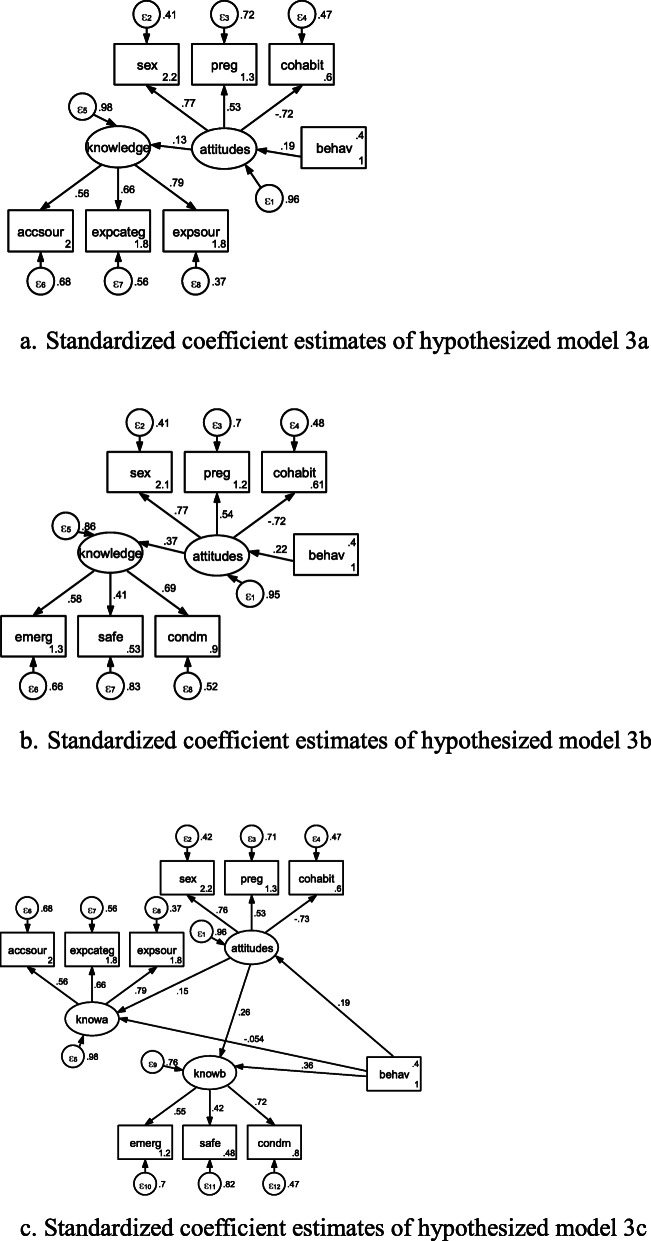
**a**. Standardized coefficient estimates of hypothesized model 3a. **b**. Standardized coefficient estimates of hypothesized model 3b. **c**. Standardized coefficient estimates of hypothesized model 3c

**Fig. 4:**
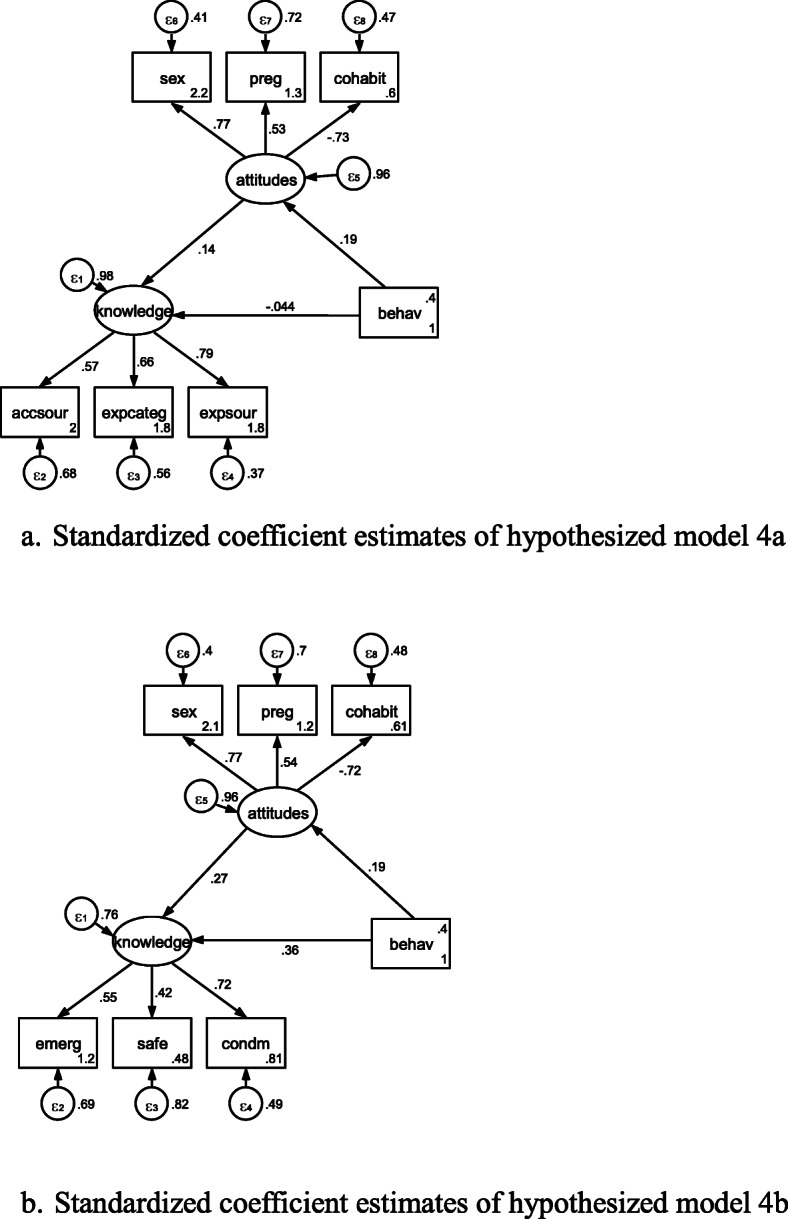
**a**. Standardized coefficient estimates of hypothesized model 4a. **b**. Standardized coefficient estimates of hypothesized model 4b.

Thus, hypothesized Figures 3 and 4 could be depicted as follow:


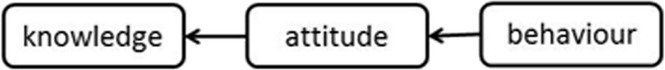


Hypothesized Figure 3. Linear sequence model in reverse direction.


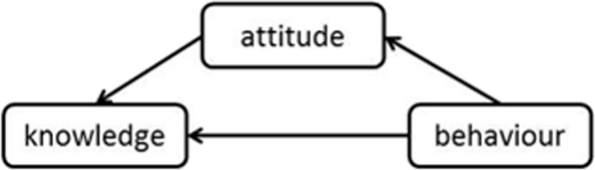


Hypothesized Figure 4. Triangle mediating model in reverse direction.

## Methods

### Data source

Data adopted here were from Survey on the Status Quo of Family Planning Management and Reproductive Health Cognition in College Students (in Chinese: 高校学生计划生育管理与生殖健康认知现状调查, http://cnsda.ruc.edu.cn/index.php?r=projects/view&id=76571348) in Beijing in 2010-2011 funded by Institute of health research, School of Social and Population Studies, Renmin University of China. These data have been published by Chinese National Survey Data Archive (CNSDA, http://www.cnsda.org/). CNSDA is an economic and social data sharing platform funded by Key projects of National Natural Science Foundation of China and implemented by National Survey Research Center at Renmin University of China. Anyone can download the data after registration. The survey questionnaire covered sociodemographic information, sources and categories of SRH knowledge, and health seeking behaviours.

### Measures

#### Sociodemographic factors

Here, sociodemographic factors included age group (15-18 and ≥ 19), gender (female and male), and educational level (undergraduate level: freshman, sophomore, junior, and senior; postgraduate level: master’s candidates and Ph. D candidates).

Educational duration in China enlightened the author to categorize the age groups. Starting ages in education system in China are generally distributed by 7 years old in primary school (educational time: 5-6 years), 13 years old in junior secondary school (educational time: 3-4 years), 16 years old in secondary senior school (educational time: 2-3 years), and 19 years old in bachelor's degrees (educational time: 4-5 years). Without compulsory starting age, postgraduate education often initiates after undergraduate education. On the basis of sample characteristics, age groups (15-18, 19-23, and 24-38) were coarsely categorized.

#### Sources and categories of SRH knowledge

The variables, accessible knowledge sources, expected knowledge categories, and expected knowledge sources were from three questions, respectively. The first question was: “Which of the following sources do you gain SRH knowledge?” The response options were advertisement leaflets, medical personnel, health educators, school students and friends, TV and magazines, instruction and curriculum, internet, and others. The second question was: “What was expected SRH knowledge of university students?” The response options were sex psychology, venereal disease, contraception, eugenics, sexual behavior, sex ethics, bisexual relationship, birth policy, and genital anatomy. The third question was: “What channels do you want to acquire SRH knowledge?” The response options were advertisement leaflets, lecture series, health educators, medical personnel, school students and friends, TV and magazines, instruction and curriculum, face-to-face consultations, internet, and others. Thus, accessible knowledge sources, expected knowledge categories, and expected knowledge sources could be obtained by summarizing the number of response options, respectively.

#### Categories of contraceptive knowledge

Categories of contraceptive knowledge were reflected by three questions: “Do you have the knowledge of emergency contraception?”, “Do you have the knowledge of safety period?”, and “Do you have the knowledge of condom use?” Their response options were binary values (no = 0, yes = 1).

#### Sexual Attitudes

Sexual attitudes included attitudes towards unmarried sex, unmarried pregnancy, and unmarried cohabitation. The three domains were reflected by three questions: “Do you think unmarried sex is acceptable?”, “Do you think unmarried cohabitation is acceptable?”, and “Do you think unmarried pregnancy is acceptable?”, respectively. The response options of first and third questions were “accepted”, “depending on the situation”, “not accepted”, and “do not know”. For convenience, the response options were dichotomized into agreement ( =0, accepted/ depending on the situation/ do not know) and disagreement ( =1, not accepted). The response options of second question were “accepted”, “not accepted”, and “I might as well.” Similarly, the response options were grouped into agreement (=0, accepted/ I might as well) and disagreement ( =1, not accepted).

### Sexual behaviour

Sexual behaviour was reflected by the question: “Have you ever had sex?” The response options were no ( =0 ) and yes ( =1 ).

### Analysis

There were five stages in the analysis. In the first stage, the descriptive statistics with Chi-square test was used to analyze the distributions of the main variables. In the second stage, associations of sociodemographic factors (age group:, gender, and educational level) with sources and categories of SRH knowledge (accessible knowledge sources: accsour, expected knowledge categories: expcateg, and expected knowledge sources: expsour), categories of contraceptive knowledge (emergency contraception: emerg, safety period: safe, and condom use: condm), sexual attitudes (unmarried sex: sex, unmarried pregnancy: preg, and unmarried cohabitation: cohabit), and sexual behavior (behav) were explored with Poisson regressions and logistic regressions, respectively. In the third stage, generalized linear covariate measurement error models were used to analyze mediating effects of sociodemographic factors on the associations between sources and categories of SRH knowledge and sexual behavior, the associations between categories of contraceptive knowledge and sexual behavior, and the associations between sexual attitudes and sexual behavior with Stata program “cme” [33]. In the fourth stage, the mediating effects of observed sexual attitudes on the associations of sources and categories of SRH knowledge and categories of contraceptive knowledge with sexual behavior were explored with 4-way decomposition using parametric regression models (4-wayDPRM) (Stata programme “med4way”) [34]. The 4-wayDPRM with delta method standard errors included logistic regression model for the outcome variable (sexual behavior), logistic regression model for the mediators (sexual attitudes: unmarried sex, unmarried pregnancy, unmarried cohabitation), exposure variables (sources and categories of SRH knowledge (knowa) and categories of contraceptive knowledge (knowb)), and covariates (sociodemographic factors: age group, gender, and educational level). In the final stage, structural equation models (SEMs) were conducted to explore mediating effects of latent sexual attitudes on the basis of the hypothesized relationships among SRH knowledge, sexual attitudes, and sexual behaviour.

The goodness-of-fit for the SEMs with categorical data were determined by indices cutoff criteria: ratio of Chi-square to the number of free parameters (χ^2^:df ratio < 2 good [35]; < 3 permissible [36]), Tucker-Lewis index (TLI) (≥0.96), comparative fit index (CFI) ( ≥ 0.95), Standardized Root Mean Squared Error (SRMR) (< 0.08 [37, 38]), Root Mean Squared Error of Approximation (RMSEA) ( < 0.05: good, 0.05-0.08: adequate, > 0.1: poor) and p of Close Fit (PCLOSE) (> 0.05 [39]), and coefficient of determination (CD). These assessment criteria could be adopted in large sample size ( > 1000) [40]. Here, mediational analysis (including observed and/or latent variables) was conducted with Stata package medsem [41]. Analysis was conducted in Stata 14.0 for Windows (Stata Corp, College Station, TX, USA).

## Results

The total sample consisted of 1196 respondents. In table 1, basic sample characteristics of university students were reported. The mean age of respondents was 20.75 years (standard deviation = ± 1.91 years) ranging from 15.94 to 37.89 years. Meanwhile, most of the sample was undergraduates (87.78%) and females (50.63%). Simultaneously, the respondents had limited knowledge of emergency contraception (61.82%), safety period (76.82%), and condom use (54.04%). In the meanwhile, part of them accepted unmarried sex (32.77%), unmarried cohabitation (60.05%), and unmarried pregnancy (18.72%). Accordingly, insufficient sexual knowledge and unfavorable sexual attitudes were observed in this sample.

**Table 1:** Sample characteristics by gender in Beijing, China in 2010-2011

	Gender		Chi square	*P* value
Age group (N=1,100)	Male (%)	Female (%)	17.4647	0.000***
15-18	10.73	5.73		
≥19	40.27	43.27		
Educational level (*N*=1,186)			9.4684	0.002***
Postgraduate	4.72	7.50		
Undergraduate	45.87	41.91		
Accessible knowledge sources, median (IQR)	3(2)	3(2)		
Expected knowledge categories, median (IQR)	3(3)	4(3)		
Expected knowledge sources, median (IQR)	3(2)	3(2)		
Knowledge of emergency contraception (*N*=1,176)			13.3200	0.000***
No	22.19	15.99		
Yes	29.17	32.65		
Knowledge of safety period (*N*=1,169)			6.2467	0.012**
No	41.23	35.59		
Yes	10.44	12.75		
Knowledge of condom use (*N*=1,175)			134.7675	0.000***
No	36.17	17.87		
Yes	15.15	30.81		
Unmarried sex (*N*=1,187)			32.9291	0.000***
Agreement	11.79	5.31		
Disagreement	38.84	44.06		
Unmarried pregnancy (*N*=1,186)			22.8226	0.000***
Agreement	22.43	15.26		
Disagreement	28.16	34.15		
Unmarried cohabitation (N=1,184)			47.3392	0.000***
Agreement	34.80	42.06		
Disagreement	15.96	7.18		
Sexual behaviour (*N*=1175)			72.2138	0.000***
No	47.91	37.87		
Yes	2.89	11.32		

Note: *, **, *** denote significance at 10%, 5%, and 1% levels, respectively. *IQR* interquartile range

With respect to accessible knowledge sources, the majority of sample (N=1191) obtained SRH knowledge from internet (57.60%), followed by schoolmates and friends (54.24%), TV and magazines (53.57%), advertisement leaflets (40.39%), instruction and curriculum (35.10%), health educators (33.33%), and medical personnel (20.49%).

Regarding knowledge categories, the majority of sample expected to obtain knowledge of sexual mental health (64.01%, *n* = 1,167), followed by relationship between two sexes (57.24%, *n* = 1,167), contraception knowledge (55.70%, n = 1,167), normal sexual behaviour (54.93%, *n* = 1,167), prevention from STIs (51.59%, *n* = 1,167), eugenics (42.84%, *n*=1,167), sexual ethics (31.79%, *n* = 1,167), childbearing policy (21.17%, *n* = 1,167), and genital anatomy (15.09%, n=1,166).

Considering expected knowledge sources, the majority of sample (*N* = 1168) expected to obtain SRH knowledge from series of lectures (44.69%), followed by health educators (40.24%), internet (44.09%), instruction and curriculum (35.87%), TV and magazines (33.65%), medical personnel (33.30%), advertisement leaflets (27.31%), schoolmates and friends (22.17%), and face-to-face consultation (18.07%).

Table 2 reported those students aged ≥19 years were >1 times more likely to have sources and categories of SRH knowledge, knowledge of emergency contraception, and favorable attitudes towards unmarried sex and unmarried pregnancy than those students aged 15-18 years. They were also <1 times less likely to have knowledge of safety period, favorable attitude towards unmarried cohabitation, sexual behaviour than those students aged 15-18 years. Females tended to have more sources and categories of SRH knowledge, knowledge of emergency contraception and condom use, attitudes towards unmarried sex and unmarried pregnancy, and sexual behaviour and less favorable attitude towards unmarried cohabitation when compared to males. Meanwhile, undergraduates tended to have more sources and categories of SRH knowledge, less knowledge of safety period and condom use, more favorable attitude towards unmarried sex, and less sexual behaviour as compared to postgraduates.

**Table 2:** Incidence rate ratios and odds ratios of sources and categories of sexual and reproductive health knowledge, categories of contraceptive knowledge, sexual attitudes, and sexual behavior in Beijing, China in 2010-2011

	sources and categories of SRH knowledge, IRR(95%CI)			categories of contraceptive knowledge, sexual attitudes, and sexual behavior ,OR (95%CI)						
	accsour	expcateg	expsour	emerg	safe	condm	sex	preg	cohabit	behav
Age group										
15-18	1 [Reference]	1 [Reference]	1 [Reference]	1 [Reference]	1 [Reference]	1 [Reference]	1 [Reference]	1 [Reference]	1 [Reference]	1 [Reference]
≥19	1.544*** (1.412-1.687)	1.591*** (1.466-1.726)	1.546*** (1.414-1.690)	1.421*** (1.096- 1.844)	0.583*** (0.441-0.771)	1.040 (0.792-1.367)	2.221*** (1.641-3.008)	1.259* (0.971-1.633)	0.452*** (0.340-0.601)	0.526*** (0.377- 0.733)
Gender										
Male	1 [Reference]	1 [Reference]	1 [Reference]	1 [Reference]	1 [Reference]	1 [Reference]	1 [Reference]	1 [Reference]	1 [Reference]	1 [Reference]
Female	1.129*** (1.053-1.210)	1.227*** (1.153-1.305)	1.207*** (1.125-1.295)	1.485*** (1.162-1.897)	1.197 (0.908-1.579)	3.692*** (2.867-4.753)	2.627*** (1.882-3.665)	1.698*** (1.330-2.167)	0.390*** (0.291-0.523)	2.603*** (1.829-3.704)
Educational level										
Postgraduate	1 [Reference]	1 [Reference]	1 [Reference]	1 [Reference]	1 [Reference]	1 [Reference]	1 [Reference]	1 [Reference]	1 [Reference]	1 [Reference]
Undergraduate	1.944*** (1.787-2.115)	2.403*** (2.223-2.596)	1.881*** (1.728-2.047)	1.019 (0.801-1.297)	0.410*** (0.318-0.530)	0.424*** (0.327-0.550)	1.772*** (1.348-2.330)	1.029 (0.809-1.308)	0.894 (0.688-1.161)	0.131*** (0.096-0.179)
Observations	1,088	1,066	1,068	1,074	1,068	1,073	1,085	1,083	1,083	1,074

Note: *, **, *** denote significance at 10%, 5%, and 1% levels, respectively. IRR= incidence rate ratio. OR =Odds Ratio. CI= confidence interval.. accsour= accessible knowledge sources, expcateg= expected knowledge categories, expsour= expected knowledge sources, emerg= knowledge of emergency contraception, safe= knowledge of safety period, and condm= knowledge of condom use , sex= attitude towards unmarried sex, preg= attitude towards unmarried pregnancy, and cohabit= attitude towards unmarried cohabitation, and behav= sexual behavior

Table 3 reported mediating effects of sociodemographic factors on the associations of sources and categories of SRH knowledge and categories of contraceptive knowledge with sexual behavior. Mediating effects of sociodemographic factors on the associations between sexual attitudes and sexual behavior could be found in Supplementary Table 1.

**Table 3:** Mediating effects of sociodemographic factors on the associations between sources and categories of sexual and reproductive health knowledge, categories of contraceptive knowledge, and sexual behavior in Beijing, China in 2010-2011

	Model 1	Model 2	Model 3	Model 4	Model 5	Model 6
Outcome model						
accsour	0.059 (0.064)					
expcateg		-2.078 ** (1.002)				
expsour			-3.645** (1.438)			
emerg				-1.363 (4.885)		
safe					48.388 (36.571)	
condm						3.702 (76.517)
sociod	-5.643	265.125	276.408	241.752 (568.280)	2275.436 *** (0.437)	-12.061 (576.327)
constant	2.166*** (0.211)	-196.307*** (4.715)	-202.345***(4.883)	-187.527 (439.458)	-1860.777	4.882 (385.066)
Indirect effects of covariates via true covariate						
accsour	-0.064** (0.029)					
expcateg		2.051** (0.941)				
expsour			3.200** (1.320)			
emerg				12.794(30.317)		
safe					42.823(36.585)	
condm						-1.601(76.517)
Total effects of covariates						
accsour	-0.006 (0.057)					
expcateg		-0.026 (0.467)				
expsour			0.446 (0.631)			
emerg				11.431(29.388)		
safe					91.210(.627) 0.000	
condm						2.100(.227) 0.000
True covariate model						
sociod						
accsour	0.011** (0.005)					
expcateg		0.008** (0.004)				
expsour			0.012** (0.005)			
emerg				0.053*** (0.016)		
safe					.019(.016)	
condm						0.133 (0.015) 0.000
constant	0.699*** (0.017)	0.700*** (0.016)	0.696*** (0.016)	0.697*** (0.013)	.716	0.668 (0.010) 0.000
res. var.	0.000 (0.000)	0.003 (0.001)	0.003 (0.001)	0.002 (0.001)	.005(0.000)	0.000 (0.000)
Measurement model						
error var.	0.196 (0.005)	0.194 (0.005)	0.194 (0.005)	0.195 (0.005)	.195(.005)	0.193 (0.005)
reliability	0.000 (0.000)	0.013 (0.005)	0.013 (0.005)	0.011 (0.005)	.026(.001)	0.000 (0.000)
No. of obs	1171	1146	1149	1156	1149	1155
log likelihood	-2538.6739	-2475.2182	-2479.4629	-2472.3732	-2404.8148	-2413.6262

Note: *, **, *** denote significance at 10%, 5%, and 1% levels, respectively. sociod = sociodemographic factors, accsour = accessible knowledge sources, expcateg = expected knowledge categories, expsour = expected knowledge sources, emerg = knowledge of emergency contraception, safe = knowledge of safety period, and condm = knowledge of condom use

Controlling for confounding sociodemographic factors, the effects of mediators (sexual attitudes) on the relationship between exposure (sources and categories of SRH knowledge and categories of contraceptive knowledge) and the outcome variable (sexual behavior) could be calculated with total excess relative risk, excess relative risk due to controlled direct effect, excess relative risk due to reference interaction, excess relative risk due to mediated interaction, and excess relative risk due to pure indirect effect.

In table 4, the excess relative risk due to pure indirect effects of accessible knowledge sources → attitude towards unmarried sex, accessible knowledge sources → attitude towards unmarried cohabitation, expected knowledge categories → attitude towards unmarried cohabitation, expected knowledge sources → attitude towards unmarried cohabitation, knowledge of emergency contraception → attitude towards unmarried sex, and knowledge of safety period → attitude towards unmarried sex were significant and less than 1. The total excess relative risk of knowledge of emergency contraception → attitude towards unmarried sex, knowledge of emergency contraception → attitude towards unmarried cohabitation, knowledge of emergency contraception → attitude towards unmarried pregnancy, knowledge of safety period → attitude towards unmarried sex, knowledge of safety period → attitude towards unmarried cohabitation, knowledge of safety period → attitude towards unmarried pregnancy, knowledge of condom use → attitude towards unmarried sex, knowledge of condom use → attitude towards unmarried cohabitation, and knowledge of condom use → attitude towards unmarried pregnancy were significant and more than 1. The excess relative risk due to controlled direct effects of expected knowledge sources → attitude towards unmarried cohabitation and knowledge of safety period → attitude towards unmarried sex were significant and less than 0, while excess relative risk due to controlled direct effects of knowledge of emergency contraception → attitude towards unmarried cohabitation, knowledge of emergency contraception → attitude towards unmarried pregnancy, knowledge of safety period → attitude towards unmarried cohabitation, knowledge of safety period → attitude towards unmarried pregnancy, knowledge of emergency contraception → attitude towards unmarried cohabitation, knowledge of emergency contraception → attitude towards unmarried pregnancy were significant and more than 1. The excess relative risk due to reference interactions of knowledge of emergency contraception → attitude towards unmarried cohabitation, knowledge of safety period → attitude towards unmarried cohabitation, and knowledge of condom use → attitude towards unmarried cohabitation were significant and less than 0, while excess relative risk due to reference interactions of expected knowledge sources → attitude towards unmarried cohabitation, knowledge of emergency contraception → attitude towards unmarried sex, knowledge of safety period → attitude towards unmarried sex, and knowledge of condom use → attitude towards unmarried sex were significant and more than 0. The excess relative risk due to mediated interactions of knowledge of emergency contraception → attitude towards unmarried sex, knowledge of emergency contraception → attitude towards unmarried cohabitation, knowledge of safety period → attitude towards unmarried sex, knowledge of condom use → attitude towards unmarried sex, knowledge of condom use → attitude towards unmarried cohabitation were significant, more than 0, and less than 1.

**Table 4:** The 4-way decomposition using parametric regression models with sexual behaviour and sociodemographic factors in Beijing, China in 2010-2011 (Exposure→Mediator)

	accsour→sex	accsour→cohabit	accsour→preg	expcateg→sex	expcateg→cohabit	expcateg→preg
tereri	0.032 (0.082)	0.051 (0.076)	0.002 (0.066)	-0.037 (0.072)	0.009 (0.047)	-0.050 (0.049)
ereri_cde	0.010 (0.086)	-0.121 (0.125)	0.056 (0.067)	-0.074 ()0.150	-0.077 (0.075)	-0.092 (0.095)
ereri_intref	-0.044 (0.076)	0.102 (0.065)	-0.062 (0.061)	0.016 (0.102)	0.056 (0.042)	0.042 (0.060)
ereri_intmed	-0.004 (0.007)	-0.009 (0.006)	-0.001 (0.002)	0.001 (0.004)	-0.003 (0.002)	0.000 (0.001)
ereri_pie	0.070 ** (0.034)	0.079 ** (0.032)	0.008 (0.010)	0.021 (0.016)	0.032** (0.016)	0.000 (0.001)
	expsour→sex	expsour→cohabit	expsour→preg	emerg→sex	emerg→cohabit	emerg→preg
tereri	-0.087 (0.082)	-0.056 (0.066)	-0.061 (0.058)	2.924*** (1.066)	2.656*** (1.022)	2.916*** (1.044)
ereri_cde	-0.096 (0.201)	-0.227** (0.106)	-0.020 (0.083)	0.661 (0.822)	3.385*** (1.236)	2.278** (1.048)
ereri_intref	-0.003 (0.149)	0.13**6 (0.053)	-0.046 (0.067)	1.461** (0.671)	-1.155** (0.555)	0.495 (0.475)
ereri_intmed	0.000 (0.003)	-0.007 (0.005)	-0.001 (0.002)	0.588** (0.295)	0.334 * (0.180)	0.102 (0.103)
ereri_pie	0.012 (0.015)	0.043* (0.024)	0.006 (0.007)	0.214* (0.122)	0.093 (0.082)	0.041 (0.050)
	safe→sex	safe→cohabit	safe→preg	condm→sex	condm→cohabit	condm→preg
tereri	1.859*** (0.619)	1.985*** (0.648)	2.118*** (0.659)	4.293*** (1.396)	4.146 *** (1.388)	4.443*** (1.399)
ereri_cde	-0.435** (0.178)	2.643*** (0.853)	2.327** (0.968)	0.904 (0.965)	5.168 *** (1.663)	4.002*** (1.505)
ereri_intref	1.918*** (0.519)	-0.847* (0.448)	-0.219 (0.558)	2.807*** (1.053)	-1.651** (0.700)	0.297 (0.664)
ereri_intmed	0.290* (0.156)	0.133 (0.111)	-0.018 (0.049)	0.489** (0.250)	0.560** (0.267)	0.069 (0.155)
ereri_pie	0.085* (0.048)	0.056 (0.042)	0.028 (0.028)	0.092 (0.061)	0.069 (0.078)	0.075 (0.055)

Note: tereri=total excess relative risk; ereri_cde=excess relative risk due to controlled direct effect; ereri_intref=excess relative risk due to reference interaction; ereri_intmed=excess relative risk due to mediated interaction; ereri_pie=excess relative risk due to pure indirect effect. 4-wayDPRM=4-way decomposition using parametric regression models. accsour= accessible knowledge sources, expcateg= expected knowledge categories, expsour= expected knowledge sources, emerg= knowledge of emergency contraception, safe= knowledge of safety period, condm= knowledge of condom use , sex= attitude towards unmarried sex, preg= attitude towards unmarried pregnancy, and cohabit= attitude towards unmarried cohabitation

### Examination of the SRH knowledge → sexual attitudes → sexual behaviour Model

Standardized coefficient estimates of hypothesized models 1 and 2 could be seen in Figures 1a, 1b, 1c, 2a, and 2b, respectively. In Table 5, nearly all of standardized coefficient estimates for SEM in hypothesized models 1 and 2 were significant.

**Table 5:** Standardized coefficient estimates in hypothesized models 1 and 2 in Beijing, China in 2010-2011

	Model 1a	Model 1b	Model 1c	Model 2a	Model 2b
Structural model					
attitudes<- knowledge	0.901*** (0.008)	0.882*** (0.012)		0.901*** (0.008)	0.872*** (0.012)
attitudes<- knowa			0.783*** (0.021)		
attitudes<- knowb			0.434*** (0.034)		
Measurement model					
accsour<- knowledge	0.936*** (0.005)			0.936 *** (0.005)	
expcateg<- knowledge	0.931*** (0.005)			0.931 *** (0.005)	
expsour<- knowledge	0.945*** (0.004)			0.945 *** (0.004)	
sex<- attitudes	0.970*** (0.006)	0.959*** (0.007)	0.949*** (0.008)	0.970 *** (0.006)	0.969 *** (0.007)
preg<- attitudes	0.847*** (0.009)	0.857*** (0.010)	0.800*** (0.011)	0.847 *** (0.009)	0.852*** (0.010)
cohabit<- attitudes	0.272*** (0.029)	0.249*** (0.029)	0.233*** (0.025)	0.272 *** (0.029)	0.247*** (0.029)
behav<- attitudes	0.404*** (0.026)	0.427*** (0.025)	0.355*** (0.023)	0.404 *** (0.026)	-0.136* (0.076)
emerg<- knowledge		0.896*** (0.010)			0.885*** (0.010)
safe<- knowledge		0.578*** (0.022)			0.584*** (0.021)
condm<- knowledge		0.802*** (0.013)			0.811*** (0.013)
behav<- knowledge				0.000 (0.000)	0.620*** (0.076)
accsour<- knowa			0.931*** (0.005)		
expcateg<- knowa			0.932*** (0.005)		
expsour<- knowa			0.948*** (0.004)		
emerg<- knowb			0.863*** (0.014)		
safe<- knowb			0.602*** (0.022)		
condm<- knowb			0.830*** (0.015)		
Number of obs	1,128	1,137	1,119	1,128	1,137
Log likelihood	-10011.843	5457.5305	-12461.165	-10011.844	-5424.9874

Note: *, **, *** denote significance at 10%, 5%, and 1% levels, respectively. accsour= accessible knowledge sources, expcateg= expected knowledge categories, expsour= expected knowledge sources, emerg= knowledge of emergency contraception, safe= knowledge of safety period, and condm= knowledge of condom use , sex= attitude towards unmarried sex, preg= attitude towards unmarried pregnancy, and cohabit= attitude towards unmarried cohabitation, and behav= sexual behavior

### Examination of the sexual behaviour → sexual attitudes → SRH knowledge model

Standardized coefficient estimates of hypothesized models 3 and 4 could be seen in Figures 3a, 3b, 3c, 4a, and 4b, respectively. In Table 6, all of standardized coefficient estimates for SEM in hypothesized models 3 and 4 were significant.

**Table 6:** Standardized coefficient estimates in hypothesized models 3 to 4 in Beijing, China in 2010-2011

	Model 3a	Model 3b	Model 3c	Model 4a	Model 4b
Structural					
knowledge<-attitudes	0.879*** (0.012)	0.879*** (0.012)		0.910*** (0.011)	0.805*** (0.016)
knowledge<-behav				-0.024 (0.017)	0.168*** (0.021)
attitudes<-behav	0.402*** (0.024)	0.402*** (0.024)	0.387 *** (0.024)	0.383*** (0.024)	0.380*** (0.024)
knowa<-attitudes			0.933 *** (0.010)		
knowa<-behav			-0.039 ** (0.016)		
knowb<-attitudes			0.830 *** (0.015)		
knowb<-behav			0.154 *** (0.020)		
Measurement					
accsour<-knowledge				0.935*** (0.005)	
expcateg<-knowledge				0.930*** (0.005)	
expsour<-knowledge				0.944*** (0.004)	
emerg<-knowledge	0.895*** (0.010)	0.895*** (0.010)			0.882*** (0.010)
safe<-knowledge	0.574*** (0.022)	0.574*** (0.022)			0.577*** (0.021)
condm<-knowledge	0.799*** (0.013)	0.799*** (0.013)			0.807*** (0.013)
accsour<-knowa			0.937 *** (0.005)		
expcateg<-knowa			0.929 *** (0.005)		
expsour<-knowa			0.943 *** (0.004)		
emerg<-knowb			0.886 *** (0.010)		
safe<-knowb			0.573 *** (0.021)		
condm<-knowb			0.806 *** (0.013)		
sex<-attitudes	0.958*** (0.007)	0.958*** (0.007)	0.954*** (0.005)	0.968*** (0.006)	0.969*** (0.007)
preg<-attitudes	0.854*** (0.010)	0.854*** (0.010)	0.844 *** (0.009)	0.845*** (0.009)	0.849*** (0.010)
cohabit<-attitudes	0.246*** (0.028)	0.246*** (0.028)	0.293 *** (0.028)	0.271*** (0.028)	0.244*** (0.028)
Number of obs	1,137	1,137	1,119	1,128	1,137
Log likelihood	-5373.6336	-5373.6336	-11859.676	-9925.7291	-5341.0904

Note: *, **, *** denote significance at 10%, 5%, and 1% levels, respectively

### Assessment on SEM models’ fit and indirect effect

In Table 7, the confirmatory factor analysis of hypothesized models 1a, 2a, 2b, 3c, 4a, and 4b gave sufficient goodness-of-fit values: pclose > 0.05, χ^2^:df ratio < 3, upper bounds of RMSEA < 0.043, CFI > 0.95, TLI > 0.95, and SRMR < 0.05. Due to typographical error and the order of the variables in model 2a, 2b, and 3c, the indirect effects could not be calculated. Thus, models 1a, 4a, and 4b were considered sufficiently fit.

**Table 7:** Assessment on model fit of models 1 to 4 in Beijing, China in 2010-2011

	χ^2^:*df* ratio	RMSEA	90% CI	pclose	CFI	TLI	SRMR	CD
Model 1a	1.553	0.022	0.000-0.040	0.997	0.995	0.992	0.020	0.747
Model 1b	8.919	0.083	0.070-0.098	0.000	0.915	0.863	0.058	0.642
Model 1c	5.207	0.061	0.052-0.071	0.019	0.926	0.899	0.054	0.911
Model 2a	1.553	0.022	0.000-0.041	0.995	0.995	0.992	0.018	0.747
Model 2b	1.600	0.023	0.000-0.041	0.995	0.994	0.990	0.017	0.670
Model 3a	8.919	0.083	0.070-0.098	0.000	0.915	0.863	0.058	0.049
Model 3b	8.919	0.083	0.070-0.098	0.000	0.915	0.863	0.058	0.049
Model 3c	2.165	0.032	0.022-0.043	0.998	0.981	0.972	0.031	0.175
Model 4a	1.553	0.022	0.000-0.041	0.995	0.995	0.992	0.018	0.039
Model 4b	1.600	0.023	0.000-0.041	0.995	0.994	0.990	0.017	0.173

Note: χ^2^:df ratio = ratio of Chi-square to the number of free parameters, *TLI* Tucker-Lewis index, *CFI* comparative fit index, *SRMR* Standardized Root Mean Squared Error, *RMSEA* Root Mean Squared Error of Approximation, *PCLOSE* p of Close Fit, and CD= coefficient of determination

After calculations, indirect effect (significant estimates, confidence interval (CI), *p* < 0.001) of Delta’s test, Sobel’s test, and Monte Carlo’s test in model 1a were 0.364 (0.318, 0.411), 0.364 (0.318, 0.410), and 0.364 (0.317, 0.410), respectively. Indirect effect (significant estimates, CI, *p* < 0.001) of Delta’s test, Sobel’s test, and Monte Carlo’s test in model 1b were 0.377(0.330, 0.424), 0.377(0.332, 0.422), and 0.377 (0.331, 0.422), respectively. Indirect effect (significant estimates, CI, *p* < 0.001) of Delta’s test, Sobel’s test, and Monte Carlo’s test in model 1c were 0.278 (0.239, 0.317), 0.278 (0.240, 0.316), and 0.278 (0.240, 0.316) with the latent variable: knowa, 0.154 (0.125, 0.183), 0.154 (0.124, 0.184), and 0.154 (0.124, 0.185) with the latent variable: knowb, respectively. Indirect effect of Delta’s test, Sobel’s test, and Monte Carlo’s test in models 3a and 3b were 0.353 (0.311, 0.396), 0.353 (0.312, 0.395), and 0.353 (0.311, 0.394), respectively. Indirect effect (significant estimates, CI, *p*<0.001) of Delta’s test, Sobel’s test, and Monte Carlo’s test in model 4a were 0.306 (0.267, 0.345), 0.306 (0.267, 0.346), and 0.306 (0.266, 0.345), respectively. Due to indirect effect/total effect = 0.646, about 65 % of the effect of sexual behaviour on sources and categories of SRH knowledge was mediated by sexual attitudes. Due to indirect effect / direct effect = 1.822, the mediated effect was about 1.8 times as large as the direct effect of sexual behaviour on sources and categories of SRH knowledge. Indirect effect (significant estimates, CI, *p*<0.001) of Delta’s test, Sobel’s test, and Monte Carlo’s test in model 4b were 0.353 (0.311, 0.396), 0.353 (0.312, 0.395), and 0.353 (0.311, 0.394), respectively.

## Discussion

The sample in this study was dominated by age distribution ranging from 18 to 24 years, undergraduate level, limited knowledge of emergency contraception, safety period and condom use, gaps in sources and categories of SRH knowledge, and high prevalence of unfavorable sexual attitudes. This study confirmed that sociodemographic factors had significant associations of with sources and categories of SRH knowledge, categories of contraceptive knowledge, sexual attitudes, and sexual behavior. Subsequently, the mediating effects of sociodemographic factors on the associations of sources and categories of SRH knowledge, categories of contraceptive knowledge, and sexual attitudes with sexual behavior were confirmed. Controlling for sociodemographic factors, the effects of sexual attitudes on the relationship between sources and categories of SRH knowledge and categories of contraceptive knowledge and sexual behavior could be verified. Structural equation modeling indicated that the linear sequence of sources and categories of SRH knowledge → sexual attitudes → sexual behaviour model and the triangle mediating effects of sexual behaviour → sexual attitudes → SRH knowledge model existed.

To the best of the knowledge of the author, this was an innovative study to report the associating factors of sources and categories of SRH knowledge among the university students in the capital of China. Beijing, the capital of China, gathers over a hundred of colleges and universities. The findings in this study were in line with several early studies. For example, a cross-sectional survey was conducted at a university in Beijing reported that many students were engaging in premarital sexual intercourse [42]. Likewise, anther cross-sectional survey in Beijing reported that college students with high-risk sexual attitude and behaviors were lack of knowledge and methods [43].

Regarding sociodemographic factors, this study was in congruence with prior studies. Influenced by numerous sociodemographic factors [44], SRH knowledge of college students was mainly from schoolmates and the internet [45]. Also, sexually active adolescents obtained SRH knowledge mainly from peers or mass media rather than teachers and parents [46]. In addition, a prior study indicated SRH knowledge was related to educational attainment [47]. Regarding gender, this study was also consistent with a prior study which highlighted male students’ contraceptive responsibilities [48].

Regarding association of contraceptive knowledge with sexual behaviour, this study was in agreement with prior studies. Substantial bodies of current research showed widespread deficits in contraceptive knowledge were in pregnant adolescents [49], tertiary students [50], female undergraduates [51], and teenage mothers [52]. Even worse, medical postgraduates had poor knowledge of condom use [53]. Subsequently, poor contraceptive knowledge resulted in unprotected sexual intercourse [54]. Especially, the students with sexual experience and lack of SRH knowledge would face more risks for sexual health.

Obviously, the relationship of knowledge-attitude-behavior in this study was in line with early studies. For example, a study in public health concluded good knowledge and attitude did not positively translate into good hygiene practices [55]. Another study in medical students indicated that the knowledge could not change the oral health behaviour, but the relationship of behaviour → attitude existed [56]. A nutritional study indicated that the relationships of nutrition-related knowledge → dietary behavior, nutrition-related attitude → dietary behavior, and nutrition-related knowledge ⇄ attitude might exist [57]. But, the relationship of knowledge-attitude-behavior in this study was incongruent with an early study which reported inconformity between attitudes and behaviors and no significant correlations among attitude, behavior, and knowledge among family physicians [58].

Regarding the relationship of sexual behavior → SRH knowledge, several facts could help understanding the association. For example, a substantial proportion of out-of-school youth engaged in risky sexual behaviours without condom use in China [59]. Still, a small proportion of young adults without adequate knowledge of contraception were showing open attitude to premarital sex and engaging in risky sexual behaviours [60, 61]. Possible reasons for the relationship of sexual behavior → SRH knowledge included sexual pleasure [62], condom use experiences [63], and poor self-protection awareness [64].

### Policy implications

Considering policy intervention, this study deepened understanding the role of sexual education on the campus. Simultaneously, this study demonstrated a low level of SRH knowledge and a high proportion of unfavorable sexual attitudes in the sample. Also, some scholars argued that proper SRH education should be implemented to reduce risk sexual behaviors among college students in China [65, 66]. Thus, health education should help to enhance awareness, enrich information sources and categories, and reduce sexual risk behaviors that caused adverse outcomes.

## Conclusion

In conclusion, the effects of sexual behaviour on sexual attitudes, contraceptive knowledge, and sources and categories of SRH knowledge among the university students were demonstrated. Simultaneously, mediating effects of sexual attitudes on the associations between SRH knowledge and sexual behaviour were confirmed. Accessible sources and categories of SRH knowledge could not meet the demand of the sample. Thus, there was an urgent need for comprehensive sexual education to improve sexual behaviours and modify sexual attitudes through designing sexual education curricula for the university students.

## Supplementary Information

**Additional file 1.**

## Data Availability

http://www.cnsda.org/

## Abbreviations

SRH: Sexual and reproductive health
STIs: Sexually transmissible infections
IRR: Incidence rate ratio
OR: Odds Ratio
CI: Confidence interval
CNSDA: Chinese National Survey Data Archive
4-wayDPRM: 4-way decomposition using parametric regression models
SEM: Structural equation model
