# Correction to: Sexual and reproductive health knowledge, sexual attitudes, and sexual behaviour of university students: Findings of a Beijing-Based Survey in 2010-2011

**DOI:** 10.1186/s13690-021-00784-0

**Published:** 2022-02-28

**Authors:** Ming Guan

**Affiliations:** 1 International Issues Center, Xuchang University, Road Bayi, Xuchang, 88 Henan China; 2 Family Issues Center, Xuchang University, Road Bayi, Xuchang, 88 Henan China; 3 School of Business, Xuchang University, Road Bayi, Xuchang, 88 Henan China

**Correction to: Arch Public Health (2021) 79:215.**

**https://doi.org/10.1186/s13690-021-00739-5**

Following publication of the original article [1], due to a typesetting error, Hypothesized Figures 1, 2, 3 and 4 were missing in the article and the Funding section need to be updated.

The correct Hypothesized Figures 1, 2, 3, 4 and the updated funding section have been provided below.

The original article [1] has been updated. 
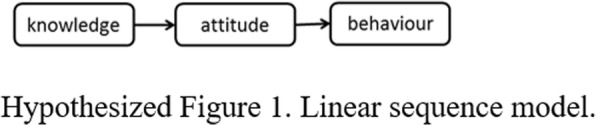

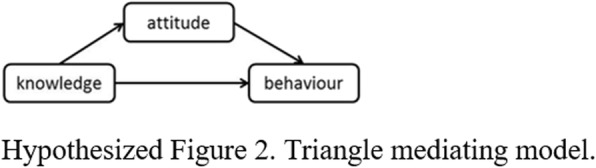

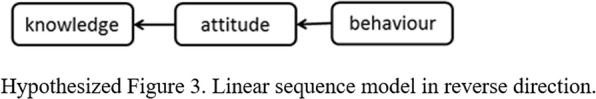

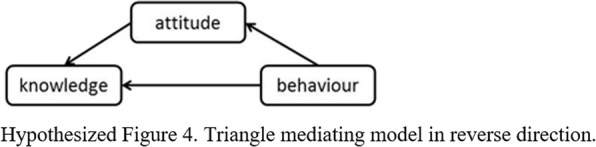


**Funding**
