# Supplementary material for: Sexual and reproductive health knowledge, sexual attitudes, and sexual behaviour of university students: Findings of a Beijing-Based Survey in 2010-2011
Source: Arch Public Health. 2021 Nov 29;79:215. doi: 10.1186/s13690-021-00739-5 (PMC8628385; doi:10.1186/s13690-021-00739-5)
Supplement: Supplementary file 1 — Additional file 1. [file 13690_2021_739_MOESM1_ESM.docx]

Supplementary Table 1. Mediating effects of sociodemographic factors on the associations between sexual attitudes and sexual behaviour

|  | Model 1 | Model 2 | Model 3 |
| --- | --- | --- | --- |
| Outcome model | | | |
| sex | 2.388*** (0.434) |  |  |
| cohabit |  | -2.018*** (0.322) |  |
| preg |  |  | 0.949 *** (0.206) |
| sociod | -4.821 | -5.289 | -5.098 |
| constant | -0.383 (0.424) | 2.417 *** (0.100) | 1.303*** (0.174) |
| Indirect effects of covariates via true covariate | | | |
| sex | -.510*** (.096) |  |  |
| cohabit |  | 0.522*** (0.095) |  |
| preg |  |  | -.288*** (.080) |
| Total effects of covariates | | | |
| sex | 1.878*** (.423) |  |  |
| cohabit |  | -1.497 *** (0.308) |  |
| preg |  |  | .661*** (.190) |
| True covariate model | | | |
| sociod |  |  |  |
| sex | 0.106 *** (0.020) |  |  |
| cohabit |  | -0.099 *** (0.018) |  |
| preg |  |  | 0.057*** (0.016) |
| constant | 0.645 *** (0.018) | 0.755 *** (0.009) | 0.697 *** (0.012) |
| res. var. | 0.000 (0.000) | 0.000 (0.000) | 0.000 (0.000) |
| Measurement model | | | |
| error var. | 0.195 (0.005) | 0.195 (0.005) | 0.196 (0.005) |
| reliability | 0.000 (0.000) | 0.000 (0.000) | 0.000 (0.000) |
| Number of observations | 1172 | 1169 | 1171 |
| log likelihood | -2513.4018 | -2506.6259 | -2528.3491 |

Note: *, **, *** denote significance at 10%, 5%, and 1% levels, respectively. sociod = sociodemographic factors, accsour = accessible knowledge sources, expcateg = expected knowledge categories, expsour = expected knowledge sources, emerg = knowledge of emergency contraception, safe = knowledge of safety period, and condm = knowledge of condom use.
